# Do Changes in Innate Immunity Underlie the Cardiovascular Benefits of Exercise?

**DOI:** 10.3389/fcvm.2019.00070

**Published:** 2019-05-29

**Authors:** Phillip Chuong, Marcin Wysoczynski, Jason Hellmann

**Affiliations:** Division of Cardiovascular Medicine, Diabetes and Obesity Center, University of Louisville School of Medicine, Louisville, KY, United States

**Keywords:** exercise, innate immnuity, cardiovascular disease, inflammation, physical activity

## Introduction

It is well established that exercise promotes health and reduces the development and progression of cardiovascular disease (CVD) (1). Traditional risk factors for CVD, such as dyslipidemia, hypertension, and diabetes mellitus, as well as all-cause mortality, are inversely correlated with cardiorespiratory fitness (2). With advancing technology and decreasing global trends in physical activity, physical inactivity is now the fourth leading cause of death worldwide (3). Furthermore, 3 million deaths per year and an estimated $53.8 billion in economic costs are lost due to insufficient physical activity (4). Although exercise promotes numerous salutary effects on the cardiovascular system, several human studies have concluded that, after controlling for reductions in traditional CVD risk factors, the beneficial effects of regular exercise are attributable to a decrease in chronic inflammation and inflammatory mediator production (1, 5–8). These clinical findings are complemented by animal studies that report regular moderate intensity exercise decreases the risk for chronic disease development through modification of the immune system (9, 10). Despite the immunoregulatory effects of exercise, the underlying cellular mechanisms and signaling pathways that promote cardiovascular health remain unknown. Identifying key determinants by which exercise modulates inflammatory responses likely offer new therapeutic targets for the treatment of cardiovascular disease. Given that the innate immune system is responsible for initiating most inflammatory responses and causally contributes to cardiac pathology and repair, we have focused this opinion manuscript on the potential role of neutrophils and macrophages in mediating the cardioprotective effects afforded by exercise.

## Exercise-Induced Changes in Innate Immunity

Neutrophils are often referred to as the first responders of the acute inflammatory response. Following activation of the endothelium and edema formation, neutrophils diapedese predominantly at postcapillary venules- and emigrate to sites of injury along chemoattractant gradients of C5a, Il-1β, TNFα, CXC chemokines (e.g., CXCL1, CXCL2, CXCL8), bioactive lipids such as leukotriene B4 (LTB_4_), and/or formylated bacteria-derived peptides. Upon phagocytosis and eradication of the inflammatory stimulus through NADPH oxidase activity, hypochlorous acid production, and/or neutralizing proteolytic enzymes, neutrophils undergo apoptosis (programmed cell death). This rapid and robust response is host protective, however in order to avoid secondary tissue damage and further propagation of the inflammatory response, apoptotic neutrophils are then cleared from the site of injury. Therefore, magnitude and duration of neutrophilic infiltration, phagocytic capacity, and subsequent apoptosis are critical factors that contribute to optimal healing. As such, mediators that prolong neutrophil survival including LPS, CRP, cyclin-dependent kinases, IL-8, GM-CSF and G-CSF delay neutrophil apoptosis and extend inflammatory responses (11, 12).

**Figure 1:**
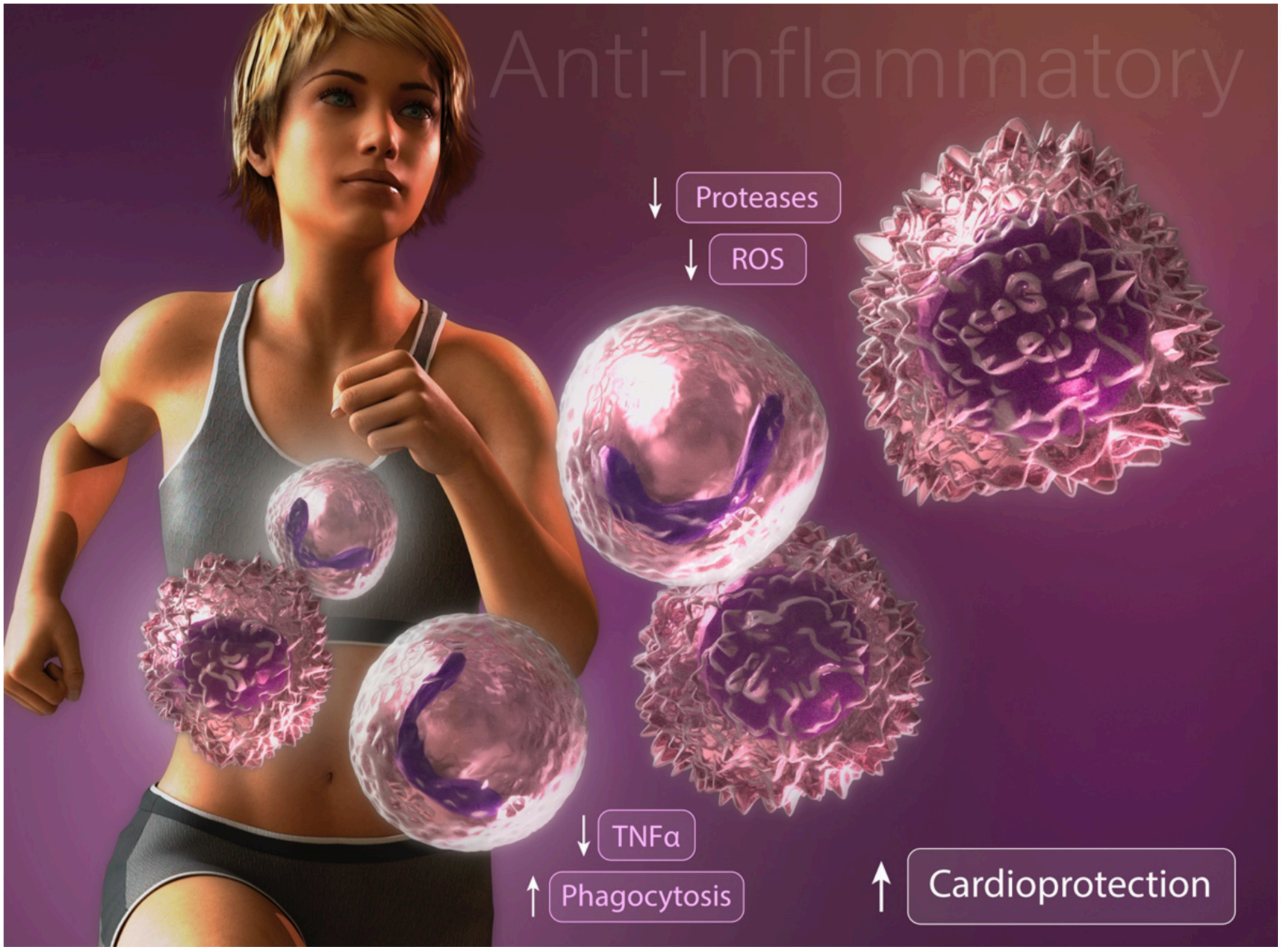
Anti-inflammatory modifications of neutrophils and macrophages may contribute to exercise-enhanced cardioprotection.

Studies focused on understanding the effects of exercise training on neutrophil function and survival have produced differing results depending on exercise intensity and duration. After a single bout of high intensity exercise, leukocyte blood counts are dramatically elevated. This rapid response is attributed to demargination of neutrophils caused by increased shear stress and the actions of catecholamines in skeletal muscle vascular pools (13–15). Furthermore, a delayed or second wave of neutrophilia develops hours later through the actions of cortisol-induced bone marrow mobilization (16). This duration and intensity dependent increase in circulating neutrophils results in increased neutrophil extravasation into skeletal muscle tissue in response to exercise-induced injury (17). Interestingly, following a single bout of exercise, unstimulated neutrophil oxidative burst, phagocytosis, and degranulation are increased whereas degranulation and oxidative burst following an inflammatory challenge are decreased (13, 18). These findings suggests that repeated exercise training alter neutrophilic function to promote an overall anti-inflammatory effect upon inflammatory challenge (Figure 1). Given their established role in propagating the inflammatory cascade during atherogenesis and myocardial ischemia, anti-inflammatory modification of neutrophils following exercise training may contribute, in part, to the exercised-induced reduction in CVD risk. Nonetheless, future studies are needed to determine the optimal exercise duration and intensity needed to promote beneficial modifications of neutrophil survival and function for CVD risk reduction.

Macrophages perform important effector and accessory immune functions and are critical mediators in the host response to tissue damage and microbial insults. A trademark feature of macrophages is their ability to engulf foreign and dying bodies (e.g., macro = big, phage = eater; *big eater*). Tissue resident macrophages actively maintain tissue homeostasis by killing microbial invaders and by non-phlogistic clearance of millions of apoptotic cells that turnover daily (19). To perform these functions, macrophages retain phagocytic, cytotoxic and anti-tumor capabilities, all of which is altered in an exercise duration and intensity dependent manner. Furthermore, during an acute inflammatory response, macrophages play a critical role in initiating the resolution phase through the phagocytosis and removal of apoptotic neutrophils to prevent collateral tissue damage caused by secondary neutrophil necrosis (20). Along these lines, macrophages play a critical role in maintaining tissue function, regeneration, and homeostasis.

In addition to tissue resident macrophages, bone marrow-derived circulating monocytes represent an immature form of macrophages, which can be rapidly recruited to sites of injury. In humans and mice, differing subsets of circulating monocytes have been described. One patrolling type of monocyte is thought to play endothelial supportive functions while another subset extravasate through the endothelium in response to inflammatory stimuli (21). This migratory process is accomplished through chemotactic gradients whereby monocytes are recruited into tissues in response to complement products, leukotrienes, IP-10, macrophage inflammatory protein (MIP), aggregated platelets, CCL2 (MCP-1), and/or CX3CL1 (fractalkine) (22). Following extravasation, monocytes mature into macrophages and, in a broad sense, differentiate into classically (in response to inflammatory challenge) or alternatively activated (to enhance pro-resolving and tissue repair) macrophages according to local tissue micro-environment conditions (23). Pro-inflammatory stimuli cause macrophages to undergo a metabolic shift away from oxidative phosphorylation and toward glycolysis (24). Classically activated macrophages are characterized by the production of inflammatory cytokines, such as Il-1ß and TNFα. Interestingly, inhibition of glycolysis using 2-deoxyglucose decreases LPS-stimulated inflammation in macrophages (25). Moreover, alternatively activated macrophages that secrete anti-inflammatory cytokines such as IL-10, rely on oxidative phosphorylation for energy production and display decreased expression of glycolytic enzymes (26).

Multiple studies have reported profound alterations in macrophage activation and function following exercise training. Consistent with an anti-inflammatory effect, high-fat diet fed mice subjected to exercise training display an enhanced proportion of alternatively activated macrophages in comparison with classically activated inflammatory macrophages in the adipose tissue (27). Furthermore, the authors report a reduction in the infiltration of inflammatory adipose tissue macrophages (27). Consistent with these findings, Kizaki et al. (28) reported that mice with free access to a running wheel have abrogated high fat diet-induced MCP-1, F4/80, and TNFα expression in adipose tissue. Importantly, these findings were recently supported in a human cohort study. Barry et al. (29) found that in the absence of weight loss, short-term moderate intensity, but not high-intensity, exercise training in obese adults resulted in down regulation of CCR2 and CXCR2 expression on circulating monocytes. Collectively, these results suggest that exercise training directly alters circulating monocytes and inhibits the infiltration of inflammatory monocyte/macrophages into adipose tissue. These data are intriguing given that inflammatory cytokine production from classically activated macrophages contributes to obesity-induced metabolic dysfunction of adipose tissue and systemic insulin resistance (30–32). These data provide one possible explanation by which exercise-induced changes promote an anti-inflammatory effect, however the underlying mechanisms remain unclear. Another possibility that may explain how exercise abrogates inflammation is found in studies assessing changes in macrophage phagocytic capacity (Figure 1). We have found that chronically inflamed obese diabetic mice display dysfunctional macrophage phagocytosis (33). Interestingly, promoting resolution of inflammation in these mice by restoring macrophage phagocytosis was associated with enhanced alternatively activated macrophage content in adipose tissue and decreased hyperglycemia, (34, 35) suggesting that dysfunctional macrophage phagocytosis may contribute to the development of insulin resistance. That exercise stimulates macrophage phagocytosis was reported several decades ago by Fehr et al (36, 37). They showed that a single bout of exhaustive endurance-running increased phagocytic activity of isolated human connective tissue macrophages and peritoneal macrophages. Others have documented an enhancement in rat peritoneal macrophage phagocytosis after just 5 min of exercise, consistent with the release of circulating factors and not alteration in phenotype (38). How duration, intensity, and diet impact exercise-induced changes in macrophage metabolic programming, phenotype, and function remains to be elucidated.

## Role of Innate Immunity in Cardiovascular Disease

Acute cardiac ischemic events such as myocardial infarction (MI), trigger a sterile systemic inflammatory response that is required for the activation of the tissue healing program (39–41). For this, increased proliferation of the bone marrow hematopoietic stem/progenitor cells and activation of extramedullary hematopoiesis in the spleen generates a surplus of myeloid cells, mostly neutrophils and monocytes (42, 43). Secreted homing factors from the infarcted myocardium results in tissue recruitment of neutrophils and monocytes for the removal of necrotic tissue, initialization of angiogenesis, and stimulation of myofibroblasts for collagen synthesis and wound healing (41). This initial myocardial myeloid response is imperative for proper healing as interventions that result in a reduction of neutrophil or monocyte/macrophage infiltration, either through systemic depletion, splenectomy, or steroid treatment, impairs optimal post-MI healing and results in excessive fibrosis and possible cardiac rupture (42, 44). On the other hand, neutrophils and monocytes/macrophages secrete pro-inflammatory mediators including proteases and reactive oxygen species that in excess negatively affect tissue healing and contribute to myocardial damage. Therefore, overproduction of myeloid cells and excessive infiltration into the ischemic myocardium may result in inappropriate inflammation, compromised tissue integrity, and formation of a non-contracting scar that alters LV pump function (45). Conversely, rats subjected to exercise training prior to permanent coronary occlusion display a reduction in myocardial collagen deposition, cardiac deterioration, and mortality (46). Chronic inflammatory diseases, however, including atherosclerosis, hypertension, diet-induced obesity, and diabetes can further contribute to overproduction of myeloid cells and cause an imbalanced myeloid response following MI and sub-optimal infarct healing (41, 45). These findings illustrate the important role of innate immunity and proper inflammatory control following MI for optimal infarct healing.

Following MI and myocyte cell death, the myocardium is besieged by an intense inflammatory response that results in the formation of a collagen-based scar (40, 41). A scar without contracting capabilities in the ischemic myocardium initiates left ventricular remodeling of the remote non-ischemic region of the myocardium, promoting hypertrophy, fibrosis, and progressive failure in pump function and the development of heart failure. Conversely, supervised exercise training in both men and women with chronic heart failure improves quality of life, reverses pathological cardiac remodeling, and decreases mortality and hospitalization above usual care (47). One of the hallmarks of patients with heart failure is chronic systemic inflammation (39–41). Pro-inflammatory cytokine levels, such as TNFα and IL-6, are closely associated with heart failure status, suggesting that inflammatory cytokine signaling contributes to progressive pump failure. Likewise, studies in mouse models of heart failure demonstrate that after MI, there is a chronically (4–8 weeks) active mononuclear phagocyte network remodeling that occurs in the spleen and non-ischemic regions of the heart (48, 49). Continuous accumulation of macrophages in the myocardium prompts progressive collagen deposition and impaired myocardial function. Inhibition of monocyte infiltration via blockade of adhesion molecules or splenectomy in mice with established heart failure dampens and improves adverse cardiac remodeling (48, 49). These findings highlight the detrimental role of an unregulated innate immune system in chronic heart failure. Interventions aimed at mitigating chronic inflammatory mediators in the post-MI myocardium offer improved outcomes in patients with ongoing pathological remodeling. Future studies are needed to determine the ideal exercise intensity and duration required for optimal cardiac remodeling.

A common cause of MI is rupture of vulnerable atherosclerotic plaques. Atherosclerosis is a chronic inflammatory disease characterized by the accumulation of lipids and leukocytes in the arterial wall. Similar to heart failure, monocytes are essential contributors to pathogenesis of the disease (50–54). Mice deficient in apolipoprotein E (ApoE KO) fed a Western style diet mobilize hematopoietic stem/progenitor cells from the bone marrow to extramedullary sites in the splenic red pulp, where they expand and differentiate to monocytes (50, 54, 55). The surplus of splenic monocytes is released into the circulation where they adhere to the atherosclerotic endothelium and extravasate into lesions and differentiate into macrophages. As a consequence of abundant uptake of low-density lipoproteins, monocyte-derived macrophages transition into foam cells. Accumulation of foam cells in arterial walls is a hallmark of early atherosclerotic lesion formation (50, 52). Continuous accumulation of monocytes and their lineage descendant macrophages can contribute to fibrous cap thickening, hematoma, thrombi, calcification, and degeneration of plaque integrity. The relative abundance of macrophages in atherosclerotic plaques is regulated by their exit and/or death, but also sustained recruitment of monocytes. Efficient macrophage exit, or reduced monocyte recruitment, results in a reduction in lesional macrophage number and regression of disease. On the other hand, factors contributing to increased monocyte recruitment accelerate atherosclerosis (56, 57). As described above, acute MI increases bone marrow and splenic myelopoiesis in response to myocardial damage. However, overproduction of monocytes due to acute MI in mice with atherosclerosis results in increased monocyte recruitment, over production of pro-inflammatory cytokines and proteolytic enzymes in the atherosclerotic lesions which stimulates growth of arterial lesions and increased risk of plaque rupture (58). Exercise promotes cardiovascular health and reduces atherosclerotic lesion size and vulnerability. In both ApoE knockout and LDL receptor knockouts fed a western style diet, aerobic exercise training reduced early lesion size formation and enhanced lesion regression (59, 60). Moreover, exercise training in diabetic ApoE knockout animals resulted in improved glucose tolerance, lesion size, and plaque stability. Interestingly, these findings were associated with decreased lesional IL-6 levels and macrophage content (61). These data suggest that the anti-atherogenic effects of exercise may relate to changes in innate immunity.

Exercise has been a hallmark treatment strategy for uncontrolled and prolonged hypertension. Recent studies demonstrate that innate immunity is involved not only in end-organ damage due to hypertension, but also in the development of hypertension. Studies in mice with Csf1 gene mutation resulting in deficiency of various subtypes of macrophages, remain normotensive, and have reduced endothelial dysfunction and vascular remodeling after Ang II infusion or DOCA-salt treatment (62, 63). Similarly, systemic depletion of monocytes using a mouse model of lysozyme M driven expression of diphtheria toxin receptor results in resistance to Ang II induced hypertension, vascular dysfunction, cardiac hypertrophy, and oxidative stress (64, 65). These data suggest that monocytes/macrophages are involved in the etiology of hypertension, but the underlying mechanism has not been fully elucidated. Furthermore, monocytes/macrophages are also involved in hypertension-induced end-organ damage. Inhibition of CCR2 receptor in hypertensive mice reduced cardiac monocyte/macrophage infiltration and attenuated hypertrophy and fibrosis (63, 65). Thus, exercise-induced changes in innate immunity may play a protective role against the development of hypertension and end-organ damage.

## Potential Role of Specialized Pro-resolving Mediators

Given their profound anti-inflammatory and pro-resolving effects on innate immune cell cytokine production and function, it is intriguing to speculate on the potential role of specialized pro-resolving lipid mediators (SPMs) in the CVD risk reducing effect of exercise. We now know that the acute inflammatory response is composed of a resolution phase that is mediated, in part, by SPMs such as lipoxins (LX), resolvins (Rv), maresins (MaR), protectins (PD), and the newly identified conjugates in tissue regeneration (e.g., RCTR, MCTR, and PCTR). SPMs are primarily synthesized from the enzymatic conversion (e.g., 5-lipoxygenase, 12-lipoxygenase, and 15-lipoxygenase) of omega 3-fatty acids and promote the resolution of inflammation by limiting excessive neutrophil infiltration, promoting apoptotic cell clearance, and enhancing overall host response (biosynthesis was recently reviewed (66, 67). Nevertheless, whether exercise affects resolution or the synthesis and actions of SPMs remains understudied. Future experiments are needed to evaluate whether systemic changes in molecular and cellular pro-resolving pathways contribute to the augmentation of innate immunity in exercise that contributes to its overall observed anti-inflammatory effect.

Produced largely by leukocytes, SPMs have been shown to have cardiovascular protective and reparative properties. Derived from EPA, RvE1 has been shown to have protective effects on cardiomyocytes and reduce infarct size in a dose dependent manner in a rat ischemia-reperfusion model (68). Additionally, RvD1 treatment reduced post-MI macrophage numbers and fibrosis resulting in a reduction in left ventricular dysfunction in mice (69). Moreover, SPMs RvE1, RvD1, RvD2, Mar1, and AT-LXA_4_ have all been shown to prevent atheroprogression and enhance lesion stability in mouse models of atherosclerosis (70–74). This exciting area of research will continue to uncover previously unknown basic mechanisms of sustained vascular inflammation and develop new therapeutic treatment options for atherosclerosis.

A limited amount of data does already exist that suggests exercise may stimulate the production of SPMs. Gangemi et al. (75) reported that urinary immunoreactive LXA_4_ levels were elevated immediately and 24 h following a single bout of strenuous maximum intensity exercise in a small healthy human cohort. These were the only data until recently when Markworth et al. (76) using targeted metabololipidomics, reported peak serum levels of LXA_4_ and LXB_4_ 1 h post-exercise, with a transient elevation in RvE1. Interestingly, the authors further reported a sustained elevation of RvD1 and PDx, a PD1 isomer, 24 h post-exercise training in humans. Consistent with these observations, Dalli et al. (77) also reported elevated plasma levels of RvD1, RvD2, and all four members of the newly identified 13-series n-3 DPA-derived resolvins, termed RvTs, in human peripheral blood samples following 30–45 min of vigorous intensity exercise. Moreover, DHA supplementation and exercise, stimulated human PBMCs to produce elevated levels of RvD1 in response to LPS. That RvD1 production is elevated in response to LPS above basal conditions following exercise was noted by the authors as a possible explanation for the overall anti-inflammatory effect of exercise (78). Collectively, these results are consistent with the idea that exercise training represents a model of self-limited sterile inflammation that requires endogenous resolution programs for repair, however whether and if exercise training alters the magnitude and duration of inflammation resolution and SPM biosynthesis is unknown.

## Future Direction

Several studies have evaluated the impact of exercise on circulating immune cell levels. Clinical studies have focused on understanding how exercise improves CVD outcomes by assessing changes in known inflammatory correlates of disease risk, including white blood cell count, IL-6, CRP, and other markers of haemostasis (e.g., fibrinogen) (1, 5, 7, 8, 79). Furthermore, preclinical investigations have uncovered basic mechanisms by which regular exercise promotes an anti-inflammatory immune response (9). Collectively, these studies have revealed that exercise reduces chronic inflammation and CVD by altering immune cell function and production of anti-inflammatory cytokines (80, 81). By describing changes in the abundance and inflammatory mediator production in circulating immune cell populations, these studies have advanced our knowledge by characterizing the anti-inflammatory effect of exercise; however questions regarding how diet and exercise intensity interact with beneficial changes in immunity remain largely unanswered. Mounting evidence suggests a critical role for SPMs in preventing or attenuating chronic cardiovascular inflammation [reviewed in (82)], however data regarding how exercise affects resolution biology is limited. We believe that future studies should continue to study changes in innate immune cell populations but that these investigations should be further expanded to incorporate assessments in cell function. A deeper understanding of how exercise alters immune cell function and the development and progression of CVD holds great therapeutic potential, especially for individuals who are often recalcitrant to exercise programs or those who could most benefit from exercise, but cannot, e.g., patients with advanced diabetes or heart failure (83–90).

## Author Contributions

All authors listed have made a substantial, direct and intellectual contribution to the work, and approved it for publication.

### Conflict of Interest Statement

The authors declare that the research was conducted in the absence of any commercial or financial relationships that could be construed as a potential conflict of interest.
